# Supplementary material for: Lifestyle change accelerates epigenetic ageing in King penguins
Source: Nat Commun. 2026 Mar 11;17:3795. doi: 10.1038/s41467-026-70527-8 (PMC13111634; doi:10.1038/s41467-026-70527-8)
Supplement: Supplementary file 1 — Supplementary Information [file 41467_2026_70527_MOESM1_ESM.pdf]

Lifestyle change accelerates epigenetic ageing in King penguins

Robin Cristofari[1]*, Leyla R. Davis[2], Gaël Bardon[3,9], Flávia A. Nitta Fernandes[1], Maria Elena Figueroa[4], Sören Franzenburg[5], Michel Gauthier-Clerc[6], Francesco Grande[4], Richard Heidrich[4], Mikaela Hukkanen[7,8], Yvon Le Maho[9], Miina Ollikainen[7,8], Elodie Paciello[9], Patrick Rampal[3], Nils C Stenseth[10], Emiliano Trucchi[11], Sandrine Zahn[9], Céline Le Bohec[3,9,12]‡ & Britta S. Meyer[13]‡

1 Institute of Biotechnology, HiLIFE, University of Helsinki, Helsinki, FI
2 Zoo Zürich, Zürich, CH
3 Centre Scientifique de Monaco, Monaco, MC
4 Loro Parque, Tenerife, ES
5 Competence Centre for Genome Analysis, Kiel, DE
6 University of Geneva, Faculty of Sciences, Geneva, CH
7 Institute for Molecular Medicine Finland, HiLIFE, University of Helsinki, Helsinki, FI
8 Minerva Foundation Institute for Medical Research, Helsinki, FI
9 IPHC/DEPE/CNRS, Strasbourg, FR
10 Centre for Ecological and Evolutionary Synthesis (CEES), Department of Biosciences, University of Oslo, NO
11 Department of Life and Environmental Sciences, Marche Polytechnic University, Ancona, Italy
12 CEFE, Université de Montpellier, CNRS, EPHE, IRD, Montpellier, FR
13 Research Unit for Evolutionary Immunogenomics, Department of Biology, University of Hamburg, 20146 Hamburg, Germany, DE

* email: robin.cristofari@helsinki.fi
‡ These authors contributed equally (in alphabetical order): Céline Le Bohec, Britta S. Meyer

Supplementary Methods

S0 | Study system and sampling

Peripheral blood was collected from 64 male King penguins at three locations: one in the wild (Crozet archipelago, see below) and two zoos (Zoo Zürich in Zürich, Switzerland and Loro Parque in Puerto de la Cruz, Spain). See Supplementary Table 1 for sample sizes and age distributions.

(1) Crozet archipelago. King penguins have been monitored in the wild using radio-frequency identification tags on Possession Island, Crozet Archipelago since 1998[1,2]. Thanks to an automated detection system, known-age individuals can be selectively recaptured at the entrance of the colony. Blood is collected periodically for a set of focal individuals, including at ~ 10 months (when the fledglings are equipped with their subcutaneous RFID tag) and at random time points through life. Birds are captured as they leave the colony, briefly restrained for morphometric measurements, and blood is collected from the brachial vein using a 23G needle. 10 µL of whole blood is stored with 700 µL Queen's Lysis Buffer and preserved at -20°C. In this study, we included repeated samples for 5 individuals at different time points (3 timepoints for 2 individuals, and 2 timepoints for 3 individuals - i.e. the 34 wild samples pertain to a total of 27 distinct individuals). For wild individuals, exact hatching date is not known, but synchronised breeding means the uncertainty is at most one month (nearly all hatching of viable chicks occurs between early January and early February) - a minor uncertainty compared to the considered lifespans.

(2) Zoo Zürich. 10 males were sampled at Zoo Zürich on May 12th, 2021. Blood was collected from the brachial vein and 10 µL whole blood were stored in 700 µL Queen's Lysis Buffer. At Zoo Zürich, King penguins are held indoors, in a light- and temperature-controlled environment, with access to a swimming area. The enclosure contains only King penguins. They are fed manually with fish, supplemented with essential nutrients such as salt and vitamins, on a daily basis, and only fast spontaneously during the moulting period. No seasonal fasting is imposed on the birds.

(3) Loro Parque. 20 males were sampled at Loro Parque on September 5th, 2022. Blood was collected from the jugular vein and 10 µL whole blood were stored in 700 µL Queen's Lysis Buffer. At Loro Parque,

King penguins are held indoors, together with other penguin species, in a photoperiod- and temperature-controlled environment, enriched with artificial snow, and access to a salt-water swimming area. Feeding is organised as in Zoo Zürich, but birds are not further supplemented in salt.

Group	N	median age	age range
Wild - Crozet	34	12	1 - 23
Zoo - Zoo Zürich	10	11	2.5 - 35
Zoo - Loro Parque	20	13	5 - 30

Supplementary Table 1 | Sample sizes, median age and range for the three sampling locations.

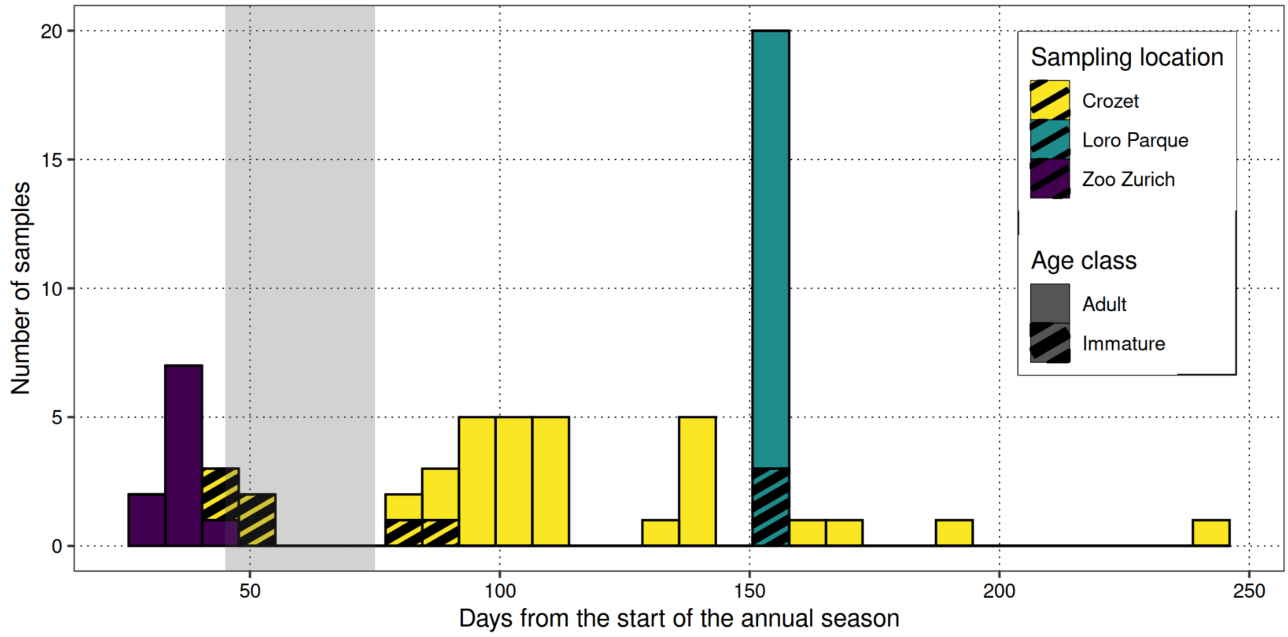

Supplementary Figure 1 | Distribution of sampling dates. To facilitate direct comparisons between zoo and wild birds, sampling dates in zoos were adjusted by six months to align with the corresponding seasonal timing in the Northern Hemisphere. Specifically, all dates are expressed relative to the start of spring—defined as October 1st for the Southern Hemisphere and April 1st for the Northern Hemisphere. In gray, we indicate the main fasting period (mid-November to mid-December in the Southern Hemisphere). Additionally, striped bars represent immature individuals (younger than 5 years) which do not engage in breeding attempts. Zoo birds do not undergo a breeding fast, hence the adult birds sampled at Zoo Zürich in spring were not fasting. Source data are provided as a Source Data file.

S1 | DNA processing and sequencing

DNA was extracted from all blood samples using a standard spin-column protocol (Macherey-Nagel Nucleospin Blood kit), including RNAse A treatment. Samples from different origins were randomly distributed across DNA extraction batches. DNA was quantified by fluorometry. Library preparation followed NEB's EMSeq kit protocol[3]. Libraries were sequenced on the Illumina NovaSeq 6000 at CCGA, Kiel, Germany. See Supplementary Table 3 for details.

S2 | Survival analysis

Given the fixed-point presence-absence nature of our dataset[2], we can strictly only estimate a right-censoring date for each individual, and not ascertain death: King penguins nearly always die at sea, so that observations of death from a land-based detection point are anecdotal. To overcome this

necessary limitation, we estimated survival status of individuals in our dataset using an empirical Bayes approach. First, we restricted data to adults (at least 5 years old) who had initiated reproduction at least once in the study colony, and had attempted reproduction at least every third year since then (effectively limiting the dataset to known breeders within the study colony to avoid the confounding effect of breeding dispersal). For these, we calculated the distribution of time intervals between two detection events.

(1) Naive classification as alive or dead. In a first step, we aimed at classifying individuals as “likely alive” or “likely dead”, by establishing the absence pattern of an individual known to be alive. To do that, we proceeded in four steps. (*i*) We established the seasonal individual discovery curve for each year between 2001 and 2021: starting at the earliest point of seasonal return in spring (15th of October), we calculated the daily number of individuals detected that day that had not yet been detected this season (Supplementary Figure 2A). (*ii*) This discovery distribution was modelled using an exponential distribution, using R package *lme4*[4], using year and day as independent variables to explain discovery rate. We underline the fact that this model is a much better representation of the right tail of the distribution (in which we are interested) than in its left tail, which is heavily influenced by the edge effect of our procedure (all birds visiting the colony regularly around the start of the period appear in our data on the first few days of discovery, leading to artifactual steepness). Overall, the fit is satisfactory (R^2 = 0.777, and Supplementary Figure 2A). Based on that distribution, we concluded that 99.9% of the individuals that would be detected this season were expected to have already been detected by January 20th. (*iii*) For each year, taking as a reference point this “full discovery date”, we calculated the time since last detection for all individuals that were known to be alive that season (i.e. that were also detected later than this reference time point) (Supplementary Figure 2A). We calculated the empirical cumulative distribution for these times since last detection, and concluded that, on average, 93.7% of individuals known to be alive on January 20th of a given year (because also detected later) had been detected within the previous 3 months, 98.8% during the previous 15 months, and 99.7% during the previous 27 months. In other words, only 0.3% birds that would later prove to be alive were ever absent from the colony for three consecutive breeding seasons (2 years and 3 months prior to the season peak on January 20th). (*iv*) Using that duration as an arbitrary threshold, we considered that a bird not detected since at least 2 years and 3 months before January 20th of the current year could be classified as “likely dead”. In this case, the bird was presumed dead during its first year of absence (since 93.7% birds return within one year to the colony) - however this is an approximation, and may lead to slightly underestimating some individuals’ lifespan.

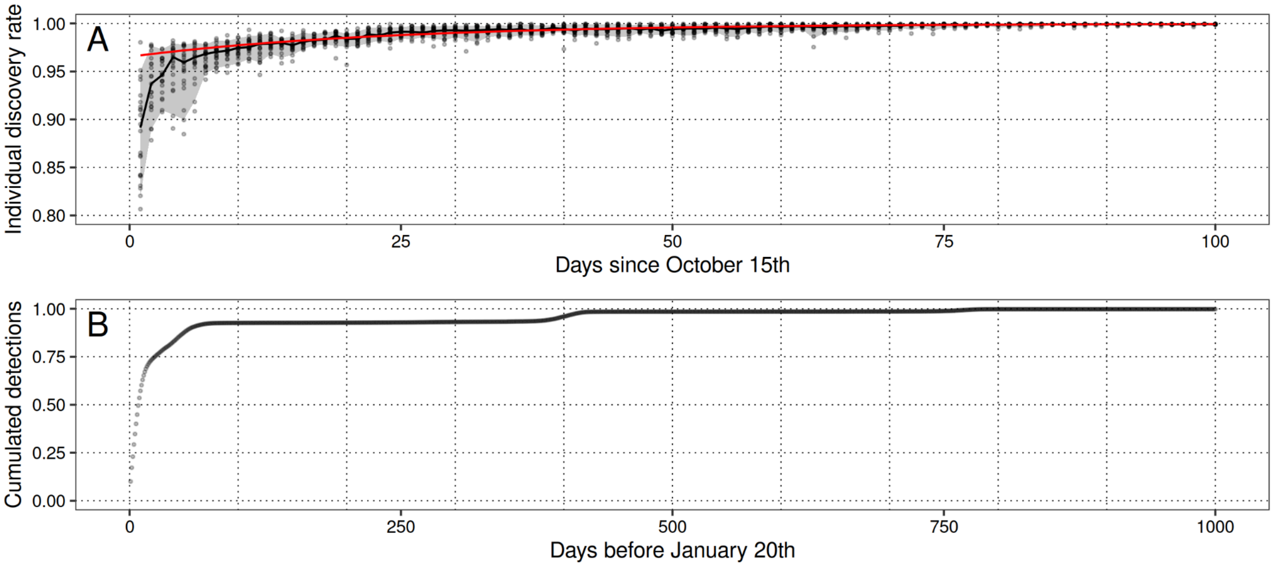

Supplementary Figure 2 | Annual re-discovery of known wild individuals through time. (A) Discovery curve for the 2001-2021 period, i.e. proportion of individuals that will breed this season, and that have already been detected on a given day, computed annually starting on Oct. 15th. Shaded, median and 90% percentile interval. In red, exponential regression line. (B) Cumulative distribution function of individual resight events, for birds known to be alive, before January 20th of a given year. > 93% of living birds have normally been seen within the 3 months before January 20th (first plateau on the curve). The remaining has been detected during the previous (second plateau), or two previous breeding seasons. The plateau structure is a consequence of the absence of most adults from the colony during winter. Source data are provided as a Source Data file.

(2) Survival modelling. Based on this classification, we estimated age- and sex-specific survival probability using a Weibull regression model, using R package *survival*[5]. Birds not seen since our classification threshold were not censored ("presumed dead"), while birds seen more recently than this threshold were right-censored ("presumed alive"). Sex and birth cohort were used as independent variables in the model.

(3) Posterior probability of being alive. In a second iteration, we calculated, for each individual, the posterior probability of being alive based on sex, age, and time interval since the last detection, using the canonical formulation of Bayes' theorem (eq. 3). (*i*) *Prior probability of being alive* (eq. 1) was the Weibull-regression predicted value, given the bird's sex and age - in other words it was the difference between age-specific survival probability at last resight, and at the reference time point (January 20th 2023). (*ii*) Probability of having been un-detected for the observed time interval given that the bird was alive (*likelihood of the "alive" model*) was derived from the observed complete distribution of inter-detection intervals, modelled using a lognormal distribution (using the "survival" package in R[5]). (*iii*) *Marginal probability of being undetected* (eq. 2) for the observed period of time was summed over both possible states, *alive* and *dead*, with the probability of the interval for a living bird given by the modelled interval distribution, and the probability of any interval for a dead bird being defined as a uniform probability density distribution between the bird's last appearance and the reference time point.

$$P_{prior}(alive) = 1 - [P(alive|AgeAtLastResight, sex) - P(alive|AgeNow, sex)] \quad (1)$$

$$P_{marginal}(I) = P(alive|age, sex) \cdot P(I|alive) + P(dead|age, sex) \cdot P(I|dead) \quad (2)$$

$$P_{posterior}(alive|I) = \frac{P(I|alive) \cdot P_{prior}(alive)}{P_{marginal}(I)} \quad (3)$$

(4) Final survival modelling. We combined data from wild and zoo individuals for the final survival analysis. We used a weighted Cox PH model, using the posterior probability of being alive as evidence weight for wild birds. Each bird is included twice: once as censored, weighted by its posterior probability of being alive, and once non-censored weighted by 1 minus its posterior probability of being alive. Weight was set to 1 for zoo birds whose death had been ascertained. We included sex and rearing environment (wild or zoo) as covariates. Results and figures are presented in the main text.

S3 | Genetic structure between groups

We tested for genetic structure between the three populations (wild birds from Crozet, and zoo birds from Zoo Zürich and Loro Parque), as epigenetic effects may be confounded by underlying genetic structure. We called single-nucleotide polymorphisms from sequencing data using the mpileup approach[6] (v. 1.16). Since cytosines are affected by the enzymatic base-conversion protocol, we retained only AT SNPs for SNP analysis. We used vcftools[7] (v. 0.1.16) to remove all sites with a phred-scaled quality below 40. Genotypes with a depth outside a range of 10X to 70X were excluded, and only sites in which all individuals were covered, and where the minor allele was observed at least 5 times, were retained, resulting in 486,501 AT SNPs. Five individuals were included at several age points in the analysis (see S0), only one of these samples was used for genetic analysis, reducing our dataset to 57 samples (27 from the wild and 30 from zoos).

We used PLINK2[8,9] (v. 2.00a3) to generate a pruned dataset containing only weakly cross-correlated loci, using PLINK's sliding-window correlation test algorithm, with a window size of 50kb by 10kb steps, and a maximum R^2 threshold of 0.1, resulting in 147,290 unlinked SNPs. These were used to compute Weir and Cockerham's pairwise F_{ST} estimator. Pairwise F_{ST} values are low between the wild and the zoo group (F_{ST} = 0.009) as well as between all three pairs of populations (Crozet / Zoo Zürich: F_{ST} = 0.022, Crozet / Loro Parque: F_{ST} = 0.013, Zoo Zürich / Loro Parque: F_{ST} = 0.034). Fixation index is very similar in Loro Parque and Crozet, with a slight excess of heterozygotes (Crozet: F = -0.045, Loro Parque: F = -0.016). There is a very slight deficit of heterozygotes in Zoo Zürich (F = 0.019).

Further, we used fastStructure[10] to explore possible population structure among the three sample groups, testing K values ranging from 1 to 5, with 10-fold internal cross-validation. Using fastStructure's model complexity choice algorithm, the single-component model (K=1) explained a cumulative ancestry contribution of > 99.99% and maximised the marginal likelihood of the model. Higher model complexities (K=2 to 5) do not suggest consistent structure between populations. Overall, we consider there is no significant population structure in our data, and that genetic variation is unlikely to confound epigenetic findings.

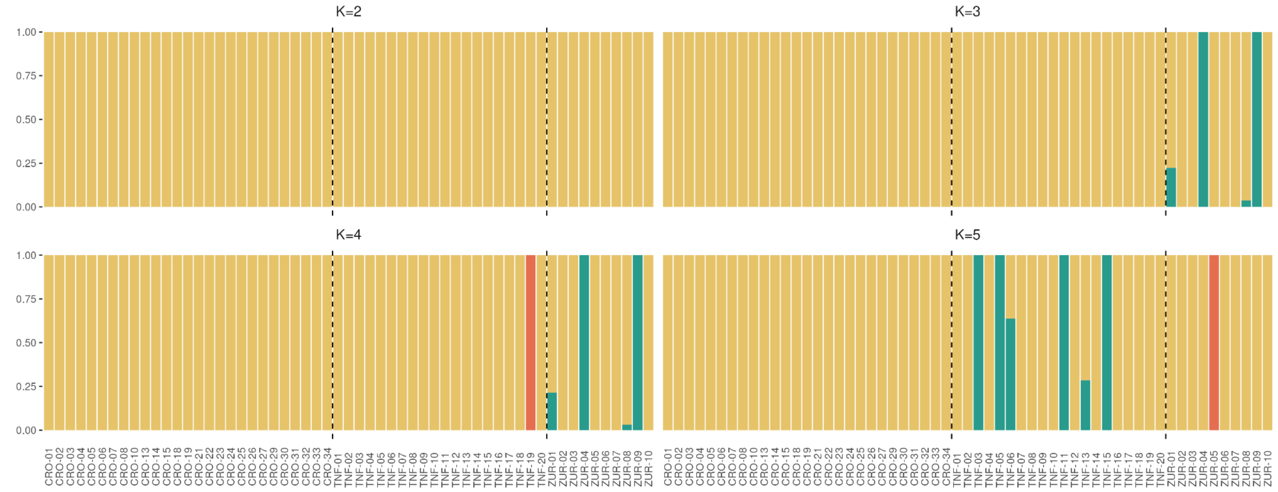

Supplementary Figure 3 | Genetic admixture inferred in fastStructure. The first panel is for K=2 components, and not one as would appear from the plot (the second admixture component has contributions of ≈0 and does not appear graphically). Source data are provided as a Source Data file.

S4 | Epigenetic age acceleration model

We chose to model epigenetic age as a linear function of time in our main methods - an approach supported by a large body of literature[11–13]. However, it is important to acknowledge that this is a simplifying assumption. There is strong evidence that epigenetic age is actually logarithmic, and not linear, with time[11,14–16]. Snir and colleagues[15] have demonstrated that this holds throughout life in humans. Because epigenetic age follows a logarithmic distribution, an increased aging rate translates mathematically into an intercept shift in log space: log(acceleration*age) = log(acceleration) + log(age). In other words, we expect an intercept, not a slope difference, between groups in the case of a slope effect on age. Of course, in that case, another expectation is that EA distribution should not be a family of straight lines, but log curves. This is true but hardly observable in empirical data: the second derivative of the log curve follows ($-1/x^2$), which means the rate of change of the local slope of the log curve becomes extremely small for larger values of x: practically, with noisy data, logarithmic distributions are approximately linear over portions of the larger end of their domain. This approximation, however, is not correct for very early life, as already underlined by Alisch and colleagues[14]. The 4 juvenile individuals included in our study would be better accounted for by a logarithmic fit, if we worked directly on average methylation values as a predictor of age. Here, we chose not to do that for two reasons: (1) the elastic net approach is more elaborate than simply averaging over methylation values with equal weights, and it is efficient at handling what is practically a local non-linearity in the data, given our sampling distribution (see Fig. 1A of the main text), and (2) we are not focusing on early life stages in this project, but rather on advanced ages: these early ages are not sampled at all at the zoo. We therefore chose to follow the approach used in the major epigenetic clock models published to date, and refined by Higgins-Chen and colleagues [13], as it has proved to be robust and largely unbiased so far.

Here, it is important to underline the distinction between, (A) on the one hand, the *linear modelling framework* used in most studies, including ours, and which approximates EA as a linear function of age over the main part of the age domain (an approach used in most published clocks) while acknowledging that this is a robust simplifying approximation and that the true underlying function is logarithmic, and (B) on the other hand, the *linear model of epigenetic ageing*, which represents ageing as an intrinsically linear process. The essential difference between these two approaches is that the first leads to expecting

differences in intercept between EAA groups, and the second would erroneously lead to expecting differences in slopes between EAA groups.

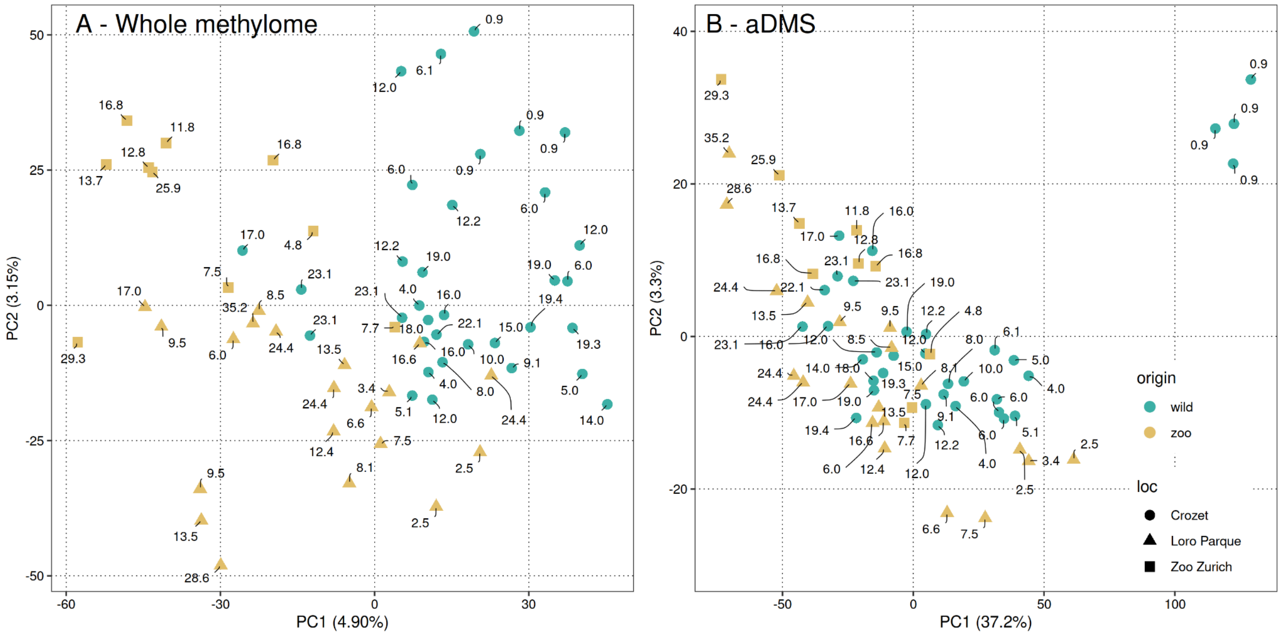

Supplementary Figure 4 | Principal component analysis of aDMS loci. A. Whole methylome, and **B.** all loci with a Pearson R^2 > 0.2. The components from this second PCA are the basis of our EA lastic net estimator. The age at sampling is indicated beside each point. Colors and shapes reflect rearing conditions and sampling location (legend in the figure). Source data are provided as a Source Data file.

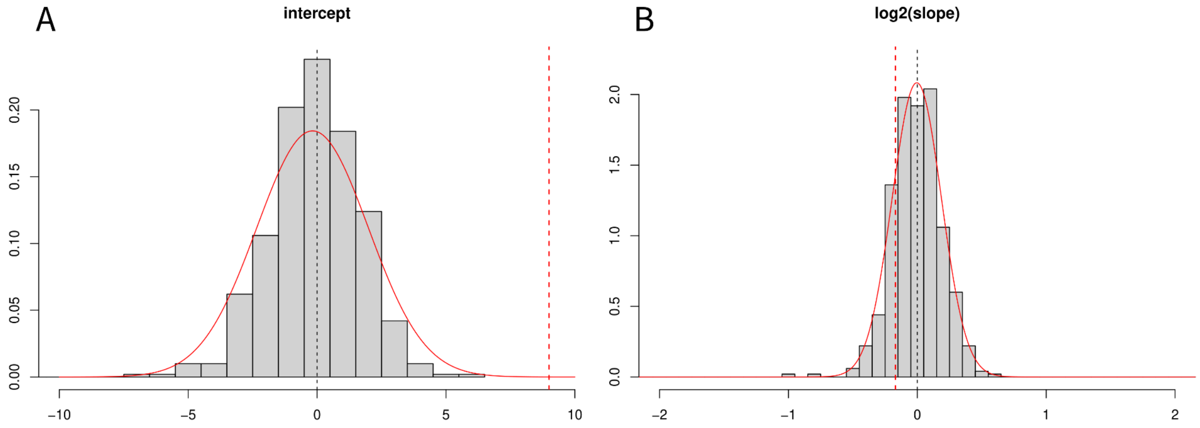

Supplementary Figure 5 | Randomisation test for elastic net model parameters. We randomised wild/zoo rearing conditions across all samples 500 times, and repeated the entire model fitting procedure for each replicate. Distribution of intercept (A) and slope (B) parameters across replicates, together with a fitted normal distribution (red curve). The parameters inferred from the original data are displayed as vertical dashed red lines. The probability of randomly observing a value at least as different from zero (two-tailed) is approximated from the normal distribution as $3.8 \times 10^{-8} \approx 0$ for the intercept, and 0.36 for the slope. This is consistent with significance estimates from linear modelling (see main text). Source data are provided as a Source Data file.

(1) *epigenetic age ~ chronological age + conversion rate + genome mean*					**AIC=361.7**
		Estimate	Standard error	t-value	p-value
	Intercept	169.572	91.960	1.844	0.0701
	Chronological age	0.849	0.064	13.321	< 2e-16 ***
	Conversion rate	-0.683	0.850	-0.803	0.425
	Genome mCpG mean	-149.686	42.763	-3.500	0.000883 ***
	Residual standard error: 3.899 on 60 degrees of freedom				
	Multiple R-squared: 0.7879, Adjusted R-squared: 0.7773				
	F-statistic: 74.31 on 3 and 60 DF, p-value: < 2.2e-16				
(2) *epigenetic age ~ chronological age + rearing group + conversion rate + genome mean*					**AIC=310.1**
		Estimate	Standard error	t-value	p-value
	Intercept (wild)	16.700	63.464	0.263	0.793
	Chronological age	0.829	0.042	19.562	< 2e-16 ***
	Group (zoo)	6.481	0.738	8.787	2.59e-12 ***
	Conversion rate	0.005	0.569	0.008	0.993
	Genome mCpG mean	-22.608	31.853	-0.710	0.481
	Residual standard error: 2.588 on 59 degrees of freedom				
	Multiple R-squared: 0.9082, Adjusted R-squared: 0.9019				
	F-statistic: 145.8 on 4 and 59 DF, p-value: < 2.2e-16				
(3) *epigenetic age ~ chronological age x rearing group + conversion rate + genome mean*					**AIC=308.7**
		Estimate	Standard error	t-value	p-value
	Intercept (wild)	46.861	64.574	0.726	0.471
	Chronological age	0.915	0.0636	14.385	< 2e-16 ***
	Group (zoo)	8.287	1.245	6.657	1.1e-08 ***
	Age x Group	-0.153	0.575	-0.404	0.687
	Conversion rate	-0.232	31.957	-1.073	0.288
	Genome mCpG mean	-34.285	0.0858	-1.784	0.0797
	Residual standard error: 2.541 on 58 degrees of freedom				
	Multiple R-squared: 0.9129,Adjusted R-squared: 0.9054				
	F-statistic: 121.6 on 5 and 58 DF, p-value: < 2.2e-16				

Supplementary Table 2 | Modelling epigenetic age as a function of chronological age. Null model (1) excludes rearing group, while models (2) and (3) include it. Additionally, model (3) includes an interaction between the epigenetic ~ chronological age regression slope, and the rearing group. Exact p-values are derived from 2-sided Student's T-tests.

Additional validations

In order to ensure our results are not an artefact of the fitting procedure, we used three independent validations strategies:

(a) In a first approach, we used the average CpG methylation at the 9,839 CpGs showing the strongest negative correlation with age in our samples, based on Pearson's correlation coefficient, without any additional covariates, with a threshold of $R^2 > 0.2$. We insist on the fact that **the selection of these loci is entirely agnostic as to the origin of the samples, which is not used at any point in locus selection / averaging - they are fully unbiased in that regard.** We then rescaled that average value to match the range of observed ages, and tested its linear correlation with the logarithm of age (panels A and B below), correcting for technical covariates (conversion rate and genome-wide methylation average). Both AIC model choice and approximate p-value agree on the significance of zoo/wild origin in explaining the resulting distribution (ΔAICwith the null model: 21.8, p-value for the "origin" factor=3.09×10^{-6}). The value of this difference is smaller than in our main model (2.6 years here, vs. 6.48 years in our main model), which is unsurprising given the much less sensitive approach used here. However, the nature and statistical significance of the result is unchanged.

(b) We applied a similar modelling approach, using the first principal component of either an aDMS (panels C and D) or a whole-methylome-wide (panels E and F) PCA regressed against log(age) as a proxy of epigenetic age. In both cases, the first PC is the one reflecting age most strongly. Here again, the results are qualitatively consistent: zoo-held King penguins appear as "age-accelerated" (for the aDMS-PCA model: AICwith the null model: 22.0, p-value for the "origin" factor=2.84×10^{-6}; for the whole-methylome model: ΔAICwith the null model: 33.8, p-value for the "origin" factor=1.07×10^{-8}). As expected, the first component of the aDMS PCA is very close to the unweighted mean methylation at aDMS - both appear to reflect the broader ageing signal across the methylome, with minor differences in CpG weighting scheme. However, we underline that in neither cases a principal component is guaranteed to covary with age (this is not e.g. an RDA), and PC1 is extremely likely to carry other signals than age.

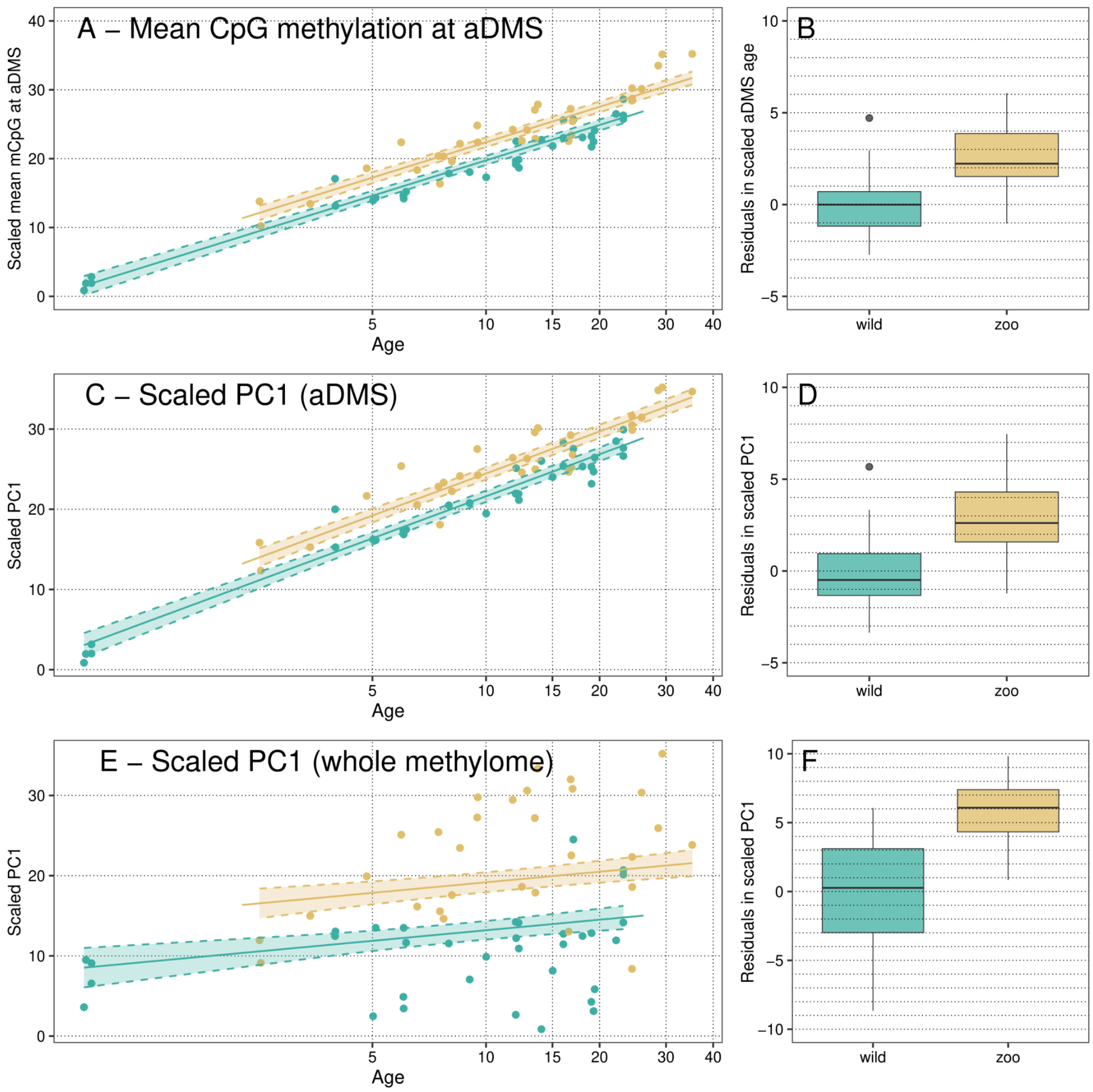

Supplementary Figure 6 | Alternative estimators of epigenetic age. Epigenetic age is estimated in three different ways: panels A and B: scaled average methylation level at aDMS sites. C and D: scaled first principal component of a PCA of all aDMS. E and F: scaled first principal component of a PCA of all genome-wide CpG sites passing quality filters. A, C and E: epigenetic age estimatoras a function of log(age), regression lines based on a linear model including rearing conditions as a two-level factor, and technical covariates (see above). B, D and F: partial residuals from these models, excluding rearing group from the predictions. Source data are provided as a Source Data file.

(c) In a third approach, we used glmnet to fit an elastic net model to our aDMS PCA coordinates, using LOOCV (as in our main analysis), but using only half of the birds to train the model: either zoo birds only (A) or wild birds only (B). Each time, the other group was predicted from that model. We show the predicted values ("Epigenetic age") regressed against the chronological age, together with the coefficients of the corresponding linear model (EA ~ age + origin). The null model is simply EA ~ age. In this case, the intercept difference for origin is fully absent from the training data, and is only inferred when regressing the predicted values against age. In both cases, estimates of age acceleration are well

supported by AIC, strongly significant, and larger than in the mean-aDMS approach. Training on zoo birds only yields a value close to our original estimate (6.00 vs 6.48 years), and training on wild birds only yields an intermediate value (3.83 years). However, while this approach provides more independent support for our conclusions, we underline the fact that it relies on a halved sample size for model training, and is likely to produce noisier coefficient values.

(d)

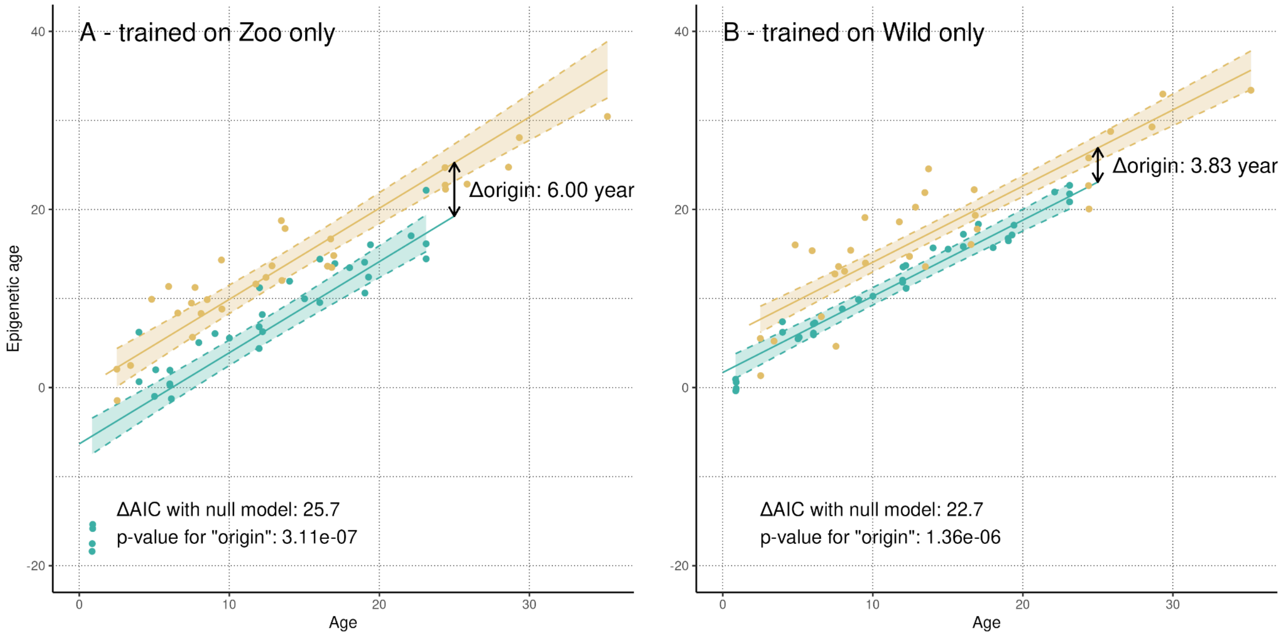

Supplementary Figure 7 | Training set validation. Epigenetic age is estimated by elastic net regression, using only zoo birds as a training and predicting for wild birds (**A**), or training on wild birds and predicting in zoo birds (**B**). The regression lines and 95%CI are derived from a linear model regressing EA against age at sampling, and rearing group. Source data are provided as a Source Data file.

(e) We applied our approach to a human test dataset of similar characteristics[17] (see Methods in the main text), contrasting a group of smokers and a group of non-smokers of known EAA. Our approach yielded results that were highly consistent with the independently-trained PCPhenoAge and PCGrimAge clocks[13]. Final mean difference between groups was 11.8 years according to our model (SE = 1.24, p-value < $1e^{-12}$), to be compared with 14.0 years according to PCPhenoAge (SE = 0.64, p-value < $1e^{-16}$) and 11.6 years according to PCGrimAge (SE = 0.57, p-value < $1e^{-16}$). Pearson's correlation coefficient between our approach and PCPhenoAge was 0.82 (R^2 = 0.67) and between our approach and PCGrimAge 0.75 (R^2 = 0.57). For reference, correlation between PCPhenoAge and PCGrimAge was 0.92 (R^2 = 0.84) - see Supplementary Figure 7C-E. The higher accuracy of the latter two clocks is expected, given they were trained on an independent sample of 1,400 individuals, as opposed to 64 here. **We underline that as in our penguin data, the application of a lifelong stressor (smoking) does not lead to diverging EAA trends between groups**, as is expected from the logarithmic model of ageing.

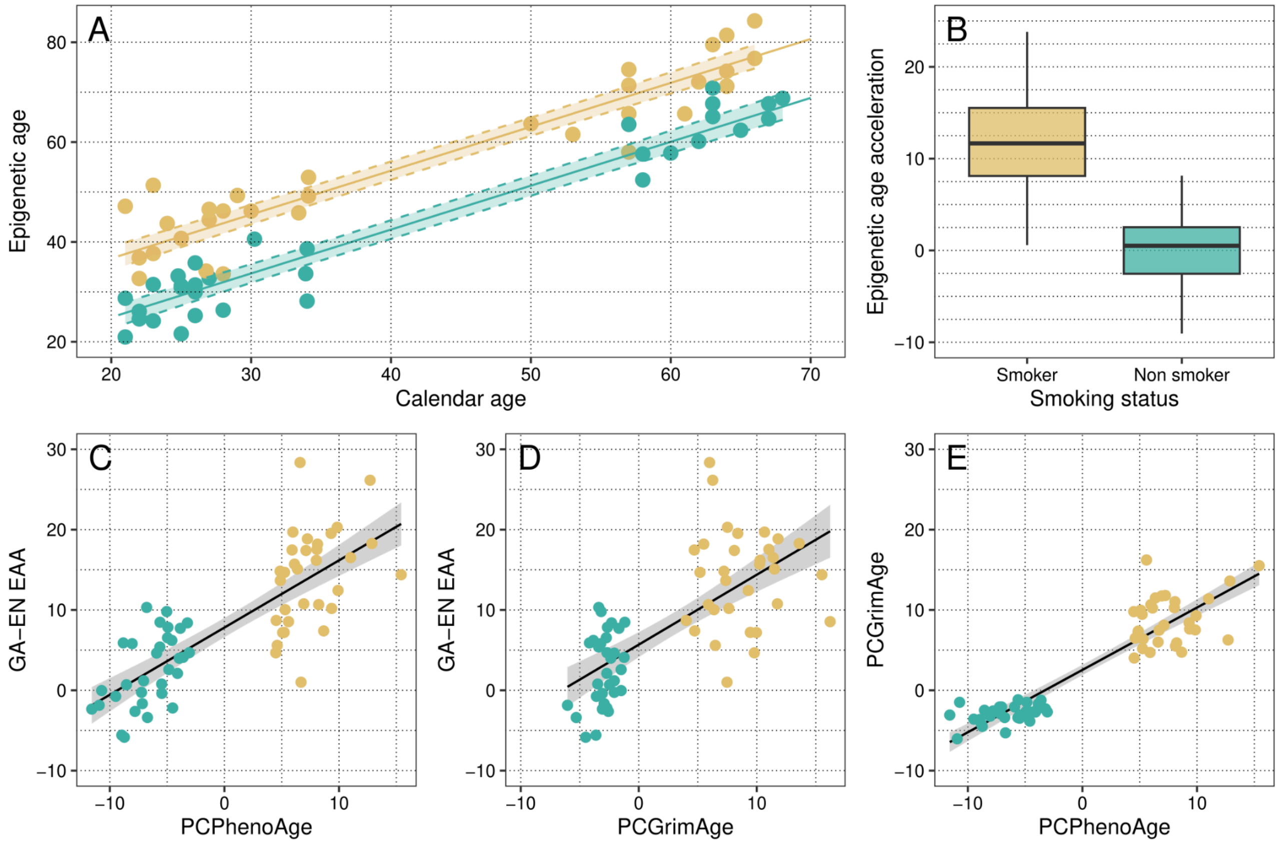

Supplementary Figure 8 | Age acceleration in smoking and non-smoking humans. (A) Epigenetic age as a function of calendar age in both groups, with 95% confidence intervals. Data points are raw age predictions based on CpG methylation. (B) Distribution of age acceleration in both groups. (C), (D) and (E): comparison between our approach (Grid-approximation - Elastic net, GA-EN on the figure) and two published epigenetic clocks for the same samples. Note that PCPhenoAge and PCGrimAge were both trained on 1,400 individuals, as opposed to 64 in our GA-EN approach. Source data are provided as a Source Data file.

S5 | Differentially methylated regions

We first evaluated CpG methylation across genomic features to validate the consistency of our dataset against prior knowledge. As expected, CpG methylation dropped sharply at the transcription start site (Supplementary Figure 8A), together with a peak in CpG density (Supplementary Figure 8B). Overall, CpG methylation was lower in CpG islands across features. It was lower in the first exon and the first exon of each gene, compared to subsequent introns-exons[18]. Genome-wide methylation level was slightly lower in zoo-reared penguins than in wild penguins, and this held globally across all features (average difference: 0.008, SE=0.0003, p-value <0.0001, linear model). Methodological artefacts could only arise from sample preservation conditions (as samples were randomised for library preparation and sequencing), and we consider these highly unlikely, as library preparation controls were not different between groups. Genome-wide average methylation level was, however, included as a covariate in all models, as this difference in background methylation rate would override any other differential methylation signal.

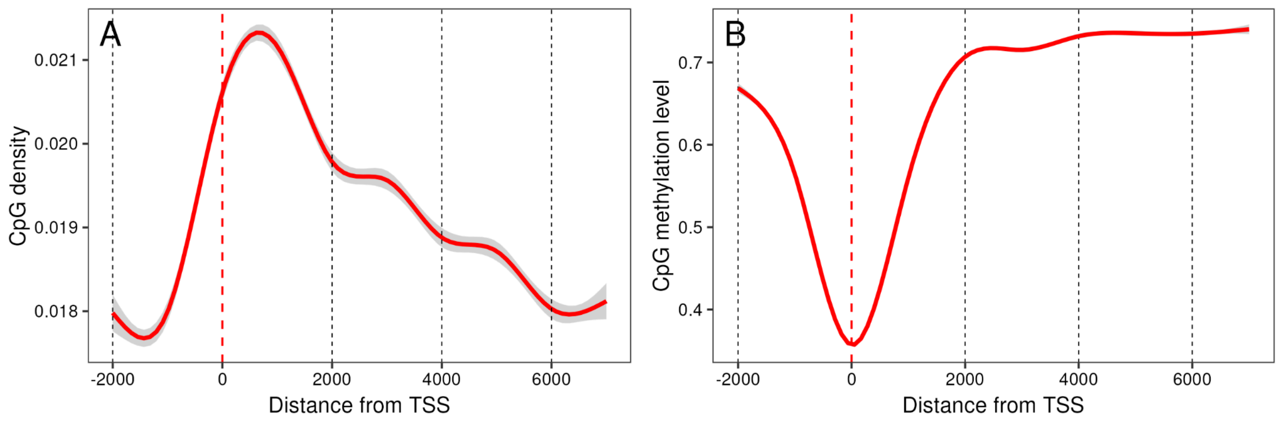

Supplementary Figure 9 | CpG density and CpG methylation around the transcription start site. (A) Average CpG density, smoothed over 250-bp sliding windows (50bp step), around the TSS. (B) Average methylation profile around the TSS. Red line: GAM smoother, with 99% confidence interval (gray area). We considered the region starting 2kb upstream of the TSS, to 7kb downstream. Source data are provided as a Source Data file.

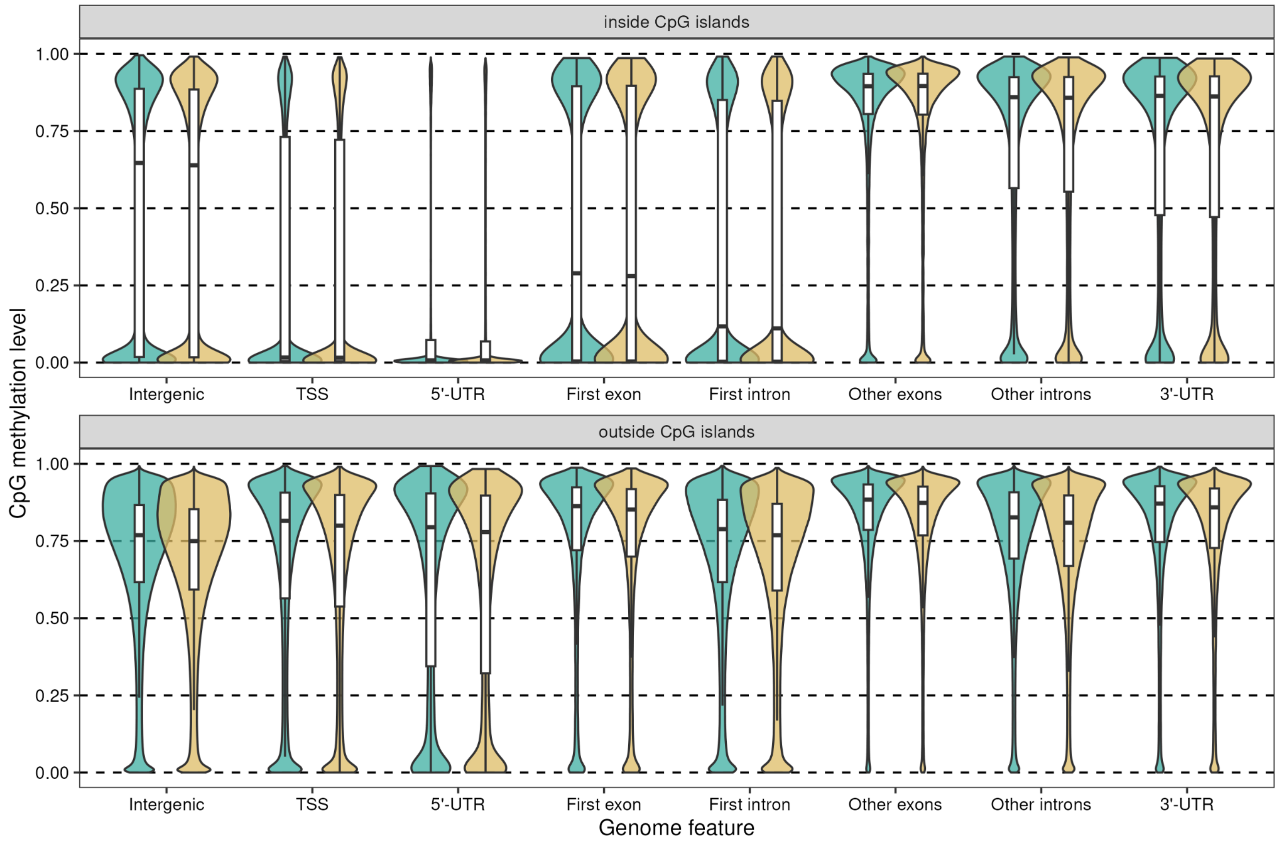

Supplementary Figure 10 | CpG methylation across genome features, inside and outside CpG islands. Source data are available on etsin.fairdata.fi (https://doi.org/10.23729/fd-e1da6857-0370-3d49-b1c3-4b7c53b366cc).

In order to evaluate the risk of false positives when using Metilene for DMR inference, we used a nested randomisation approach. First, in order to evaluate the probability of finding a false-positive DMR between random groups of birds, despite Benjamini-Hochberg FDR correction, we randomised rearing conditions across samples 500 times, and inferred DMRs based on these random assignments. We identified on average 8 DMRs in randomised data (median 7, range 0 to 40, 95% interquantile 1 to 19), to be compared with 600 DMRs inferred in the original data. Encountering a number equal or greater than

600 , under a normal distribution fitted to the random distribution, would have a probability computationally indistinguishable from zero.

Second, in order to evaluate the number of genes encountered randomly within 5kbp of a dataset of 600 DMRs (as identified in our original data), we randomly shuffled these DMRs across the genome 500 times. To account for the fact that both DMRs and genes are more likely in CpG-rich areas of the genome, we shuffled DMRs only across areas of the genome that had at least as high a GC-content as our least-GC-rich DMR (i.e. a CG content ≥ 0.33). This procedure pointed to an average of 263 genes (median 263, range 233 to 295, 95% interquantile 239 to 284), to be compared with 338 in our original DMRs. Encountering a number equal or greater than 338 , under a normal distribution fitted to the random distribution, would have a probability computationally ≤ 1e-10.

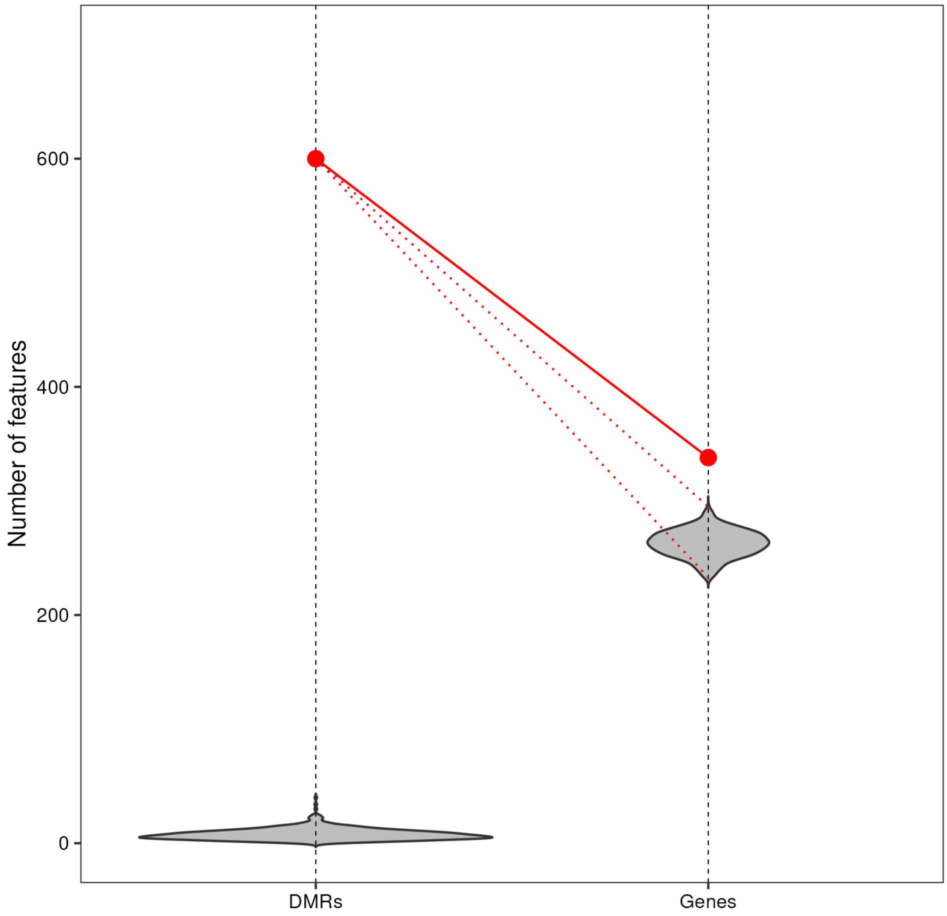

Supplementary Figure 11 | Random DMR and differentially methylated gene discovery. Violins represent random distributions based on 500 replicates. Red dots connected by a solid red line represent values inferred from the original data. The gene distribution is based on a randomisation of the 600 original DMRs (as indicated by the thin dotted lines) and not on the random-data DMRs (left violin) - in that case gene numbers are always zero. Source data are provided as a Source Data file.

Finally, we re-computed DMRs splitting zoo-reared birds in two groups according to their zoo of origin (Zoo Zürich, N=10 or Loro Parque, N=20) and computing DMRs for each pair of groups (each zoo vs wild, and Zoo Zürich vs Loro Parque). Numbers of DMRs were comparable in each case (737 DMRs for the wild vs. Loro Parque comparison, and 352 for the wild vs. Zoo Zürich comparison, compared to 600 for the wild vs both zoos comparison). Jaccard indices were high in both cases (0.52 for the wild vs. Loro Parque comparison, 0.21 for the wild vs. Zoo Zürich comparison - as opposed to 0.0021 [stdev. 0.0020] for 600

shuffled DMRs - see above for the randomisation protocol). When considering genes involved in each comparison, overlap between sets is high: 108 genes are involved in all three pairwise comparisons, and 190 in two pairwise comparisons (in all cases except 4, these were included in the full-set Wild vs. Zoo DMRs). Finally, 242 genes were only involved in one pairwise set of DMRs - of these possible false-positives, only 44 were uniquely found in the full Wild vs. Zoo DMRs. More noise is expected in the reduced sets, due to the reduced sample size. We consider these results to show our DMR inference is clearly out of the random expectation, and retain the full-set result for further analysis.

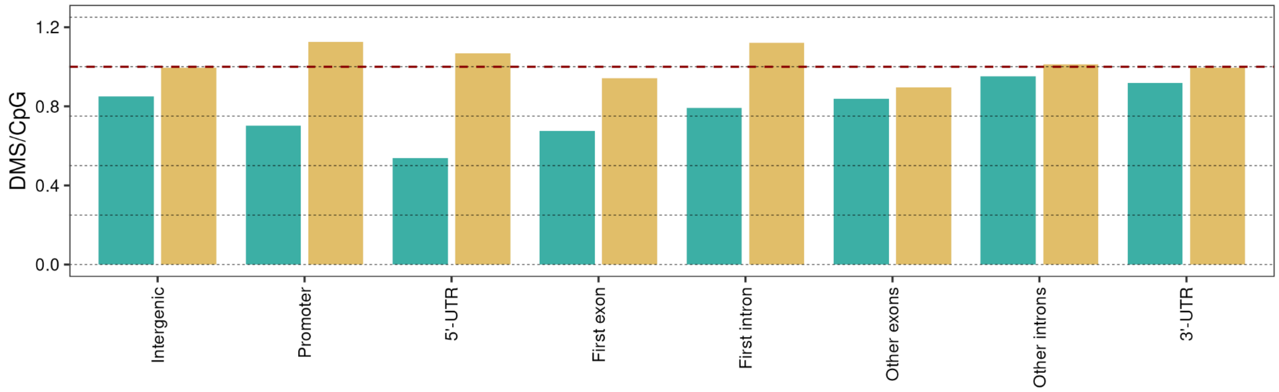

Supplementary Figure 12 | DMS density across genome features, scaled to genome-wide DMS density. Inside CpG islands in green (left bars) and outside CpG islands (tan bars, right). DMS are defined as significant binomial tests at the 0.05 level, taking all relevant covariates into account (see main methods). Overall, we observe less DMS in CpG islands, and especially so in CpG-rich promoters, 5'-UTRs and first exons, in keeping with expectations for these mostly unmethylated areas. On the other hand, there is an excess of DMS in non-CpG-island promoters, 5'UTRs and first introns - three types of features involved in gene regulation. All pairwise tests are significant, except non-CpGi non-first exons vs non-CpGi intergenic background. Source data are provided as a Source Data file.

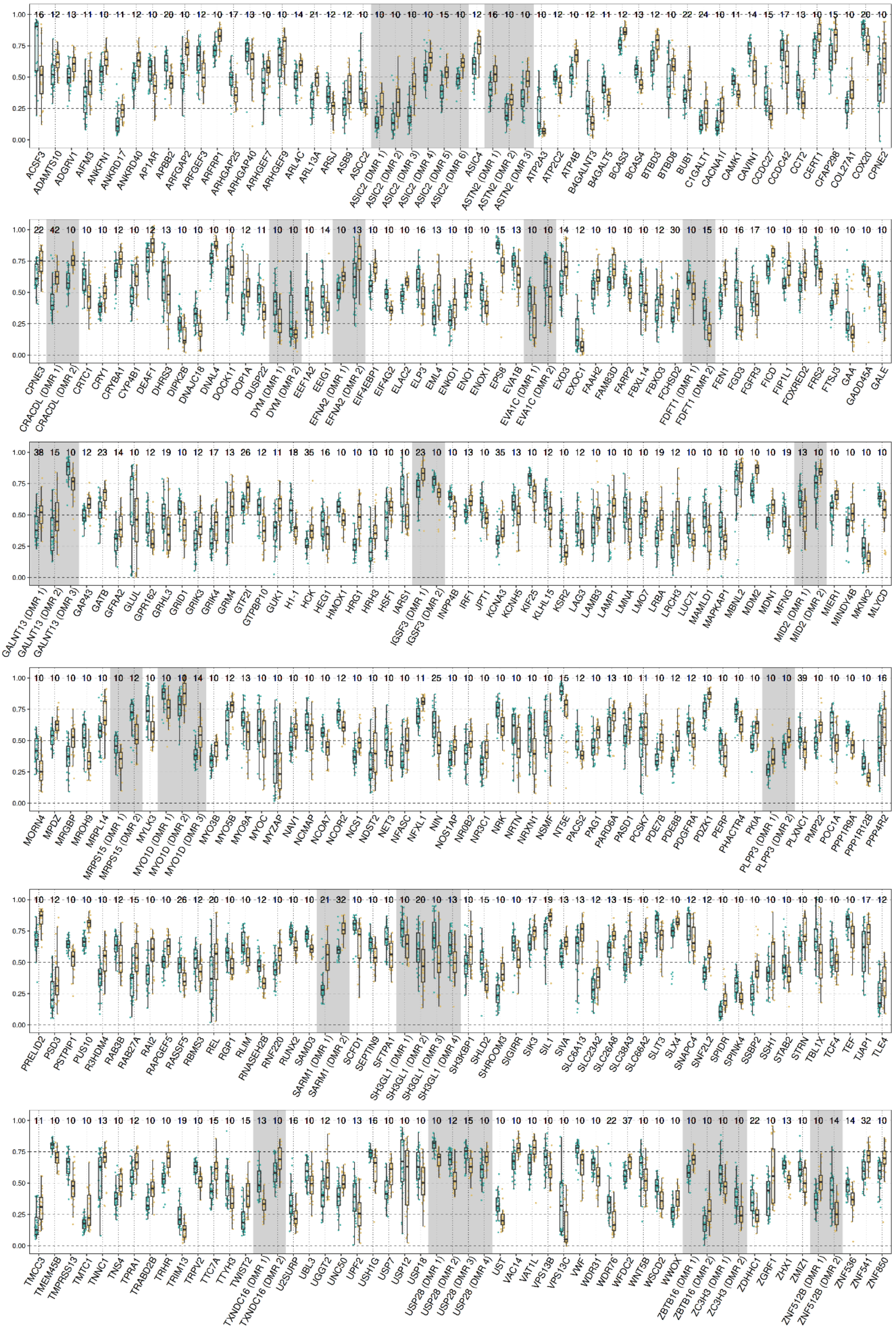

Supplementary Figure 13 | Methylation level at all genes associated with a DMR. In green (left), wild birds, in tan, zoo birds; boxplots provide color reference. Methylation levels are beta-corrected for age, EAA and genome-wide mean CpG methylation. Source data are provided as a Source Data file.

Supplementary Tables 3-4

These three tables are presented after the references section.

Supplementary Table 3 | Sequencing data processing metrics

Supplementary Table 4 | Overrepresented super-paths and pathways in genes associated with DMRs. P-values are derived from one-sided binomial tests, with Benjamini-Hochberg false-discovery correction.

References

1. Gendner, J.-P., Gauthier-Clerc, M., Bohec, C. L., Descamps, S. & Maho, Y. L. A new application for transponders in studying penguins. *J. Field Ornithol.* **76**, 138–142 (2005).
2. Bardon, G. *et al.* RFIDeep: Unfolding the potential of deep learning for radio-frequency identification. *Methods Ecol. Evol.* **14**, 2814–2826 (2023).
3. Vaisvila, R. *et al.* Enzymatic methyl sequencing detects DNA methylation at single-base resolution from picograms of DNA. *Genome Res.* **31**, 1280–1289 (2021).
4. Bates, D., Mächler, M., Bolker, B. & Walker, S. Fitting Linear Mixed-Effects Models Using lme4. *J. Stat. Softw.* **67**, 1–48 (2015).
5. Therneau, T. M. & Grambsch, P. M. *Modeling Survival Data: Extending the Cox Model*. (Springer, New York, NY, 2000). doi:10.1007/978-1-4757-3294-8.
6. Li, H. *et al.* The Sequence Alignment/Map format and SAMtools. *Bioinformatics* **25**, 2078–2079 (2009).
7. Danecek, P. *et al.* The variant call format and VCFtools. *Bioinformatics* **27**, 2156–2158 (2011).
8. Purcell, S. *et al.* PLINK: A Tool Set for Whole-Genome Association and Population-Based Linkage Analyses. *Am. J. Hum. Genet.* **81**, 559 (2007).
9. Chang, C. C. *et al.* Second-generation PLINK: rising to the challenge of larger and richer datasets. *GigaScience* **4**, 7 (2015).
10. Raj, A., Stephens, M. & Pritchard, J. K. fastSTRUCTURE: Variational Inference of Population Structure in Large SNP Data Sets. *Genetics* **197**, 573–589 (2014).
11. Horvath, S. DNA methylation age of human tissues and cell types. *Genome Biol.* **14**, 3156 (2013).
12. Bell, C. G. *et al.* DNA methylation aging clocks: challenges and recommendations. *Genome Biol.* **20**, 249 (2019).
13. Higgins-Chen, A. T. *et al.* A computational solution for bolstering reliability of epigenetic clocks: implications for clinical trials and longitudinal tracking. *Nat. Aging* **2**, 644–661 (2022).
14. Alisch, R. S. *et al.* Age-associated DNA methylation in pediatric populations. *Genome Res.* **22**, 623–632 (2012).
15. Snir, S., Farrell, C. & Pellegrini, M. Human epigenetic ageing is logarithmic with time across the entire lifespan. *Epigenetics* **14**, 912–926 (2019).
16. Bernabeu, E. *et al.* Refining epigenetic prediction of chronological and biological age. *Genome Med.* **15**, 12 (2023).
17. Kaprio, J. *et al.* The Older Finnish Twin Cohort - 45 Years of Follow-up. *Twin Res. Hum. Genet. Off. J. Int. Soc. Twin Stud.* **22**, 240–254 (2019).
18. Anastasiadi, D., Esteve-Codina, A. & Piferrer, F. Consistent inverse correlation between DNA methylation of the first intron and gene expression across tissues and species. *Epigenetics Chromatin* **11**, 37 (2018).

Supplementary Table S3

Name	Rearing group	Location	Age at sampling	Conversion rate	Genome mCpG mean	EAA	Raw reads	Filtered reads	Read retaining ratio	Median depth
A02168	wild	Crozet	0.86	97.0	0.65	-3.71	175,545,806	168,005,453	0.96	20
A03083	wild	Crozet	0.87	99.8	0.64	-4.54	267,701,608	258,812,025	0.97	31
A03374	wild	Crozet	0.90	99.8	0.64	-3.16	291,656,096	277,119,813	0.95	32
A03407	wild	Crozet	0.90	99.8	0.64	-3.19	275,622,242	263,801,300	0.96	32
KIN-BDM-2018-E-569	wild	Crozet	3.98	99.5	0.65	5.03	252,794,172	240,557,025	0.95	29
KIN-BDM-2018-E-565	wild	Crozet	3.99	99.3	0.65	1.52	250,218,998	240,728,874	0.96	29
KIN-BDM-2017-E-706	wild	Crozet	5.02	98.9	0.66	0.76	258,458,240	249,328,576	0.96	30
KIN-BDM-2017-E-654	wild	Crozet	5.10	99.4	0.65	3.17	319,410,010	306,399,248	0.96	37
A03374	wild	Crozet	6.04	99.9	0.65	-0.30	246,801,036	237,441,974	0.96	28
A03260	wild	Crozet	6.05	99.9	0.64	-2.76	217,260,290	209,041,432	0.96	25
A03407	wild	Crozet	6.05	99.8	0.64	-0.66	206,865,944	198,759,692	0.96	24
A03083	wild	Crozet	6.14	99.9	0.64	-2.30	253,956,922	243,974,665	0.96	29
KIN-BDM-2014-E-891	wild	Crozet	7.97	98.5	0.66	1.44	265,269,284	255,820,596	0.96	31
KIN-BDM-2013-E-335	wild	Crozet	9.05	99.1	0.66	0.21	277,788,362	268,005,191	0.96	32
E12630	wild	Crozet	10.01	99.0	0.66	1.34	258,707,240	249,314,895	0.96	30
A02168	wild	Crozet	11.99	99.8	0.66	1.03	225,259,472	216,098,055	0.96	26
A03083	wild	Crozet	11.99	99.9	0.64	-2.52	258,654,808	248,906,127	0.96	30
E10399	wild	Crozet	12.02	99.4	0.65	4.32	306,093,972	292,476,573	0.96	36
A00774	wild	Crozet	12.20	99.9	0.64	1.40	237,172,942	228,822,030	0.96	27
A03407	wild	Crozet	12.22	99.8	0.65	-1.60	252,319,108	241,718,595	0.96	29
E08115	wild	Crozet	14.02	98.9	0.66	0.79	175,388,374	168,658,705	0.96	20
A03354	wild	Crozet	15.02	99.4	0.65	-0.88	187,943,702	181,166,060	0.96	22
A01396	wild	Crozet	16.03	99.8	0.65	0.32	254,900,506	243,062,230	0.95	29
A01376	wild	Crozet	16.04	99.9	0.65	4.00	279,130,856	265,134,354	0.95	32
A02580	wild	Crozet	17.04	99.3	0.63	2.36	244,019,280	234,747,619	0.96	28
A01833	wild	Crozet	18.02	99.2	0.65	-0.58	243,705,328	235,469,886	0.97	29
A00985	wild	Crozet	19.02	99.8	0.65	-1.08	181,227,454	175,790,877	0.97	21
A00774	wild	Crozet	19.04	99.8	0.64	-2.34	282,460,804	270,995,333	0.96	33
A00388	wild	Crozet	19.28	99.8	0.66	-1.53	242,422,950	232,556,553	0.96	28
A00319	wild	Crozet	19.41	99.9	0.65	0.33	224,706,356	214,699,385	0.96	25
A00144	wild	Crozet	22.12	99.7	0.65	1.72	306,030,920	289,497,453	0.95	35
A00000	wild	Crozet	23.12	99.9	0.64	0.05	258,681,758	245,457,247	0.95	29
A00017	wild	Crozet	23.12	99.5	0.64	4.93	234,477,890	223,553,639	0.95	27
A00097	wild	Crozet	23.12	99.8	0.65	-3.57	245,963,864	233,260,155	0.95	28
LP97141	zoo	Loro Parque	2.51	99.6	0.65	6.43	238,569,818	229,294,907	0.96	27
LP97139	zoo	Loro Parque	2.53	99.8	0.66	2.57	235,023,396	225,947,235	0.96	27
LP97136	zoo	Loro Parque	3.42	99.2	0.65	7.00	279,156,730	266,106,892	0.95	32
LP97126	zoo	Loro Parque	5.96	99.4	0.64	10.91	265,415,408	256,597,360	0.97	31
LP97123	zoo	Loro Parque	6.57	99.6	0.65	7.18	314,165,266	298,299,779	0.95	36
LP97120	zoo	Loro Parque	7.55	99.4	0.65	4.60	238,098,072	228,997,363	0.96	28
LP97119	zoo	Loro Parque	8.12	99.7	0.65	7.23	261,761,848	251,251,262	0.96	30
LP97116	zoo	Loro Parque	8.53	99.0	0.64	8.28	361,023,376	339,187,414	0.94	41
LP97115	zoo	Loro Parque	9.48	99.3	0.63	10.96	217,395,414	209,375,696	0.96	25
LP97114	zoo	Loro Parque	9.51	99.2	0.63	6.86	233,304,916	225,130,141	0.96	27
LP97104	zoo	Loro Parque	12.45	99.6	0.65	6.15	239,248,174	230,879,525	0.97	28
LP97100	zoo	Loro Parque	13.47	99.6	0.63	12.41	250,802,280	240,250,421	0.96	29
LP97098	zoo	Loro Parque	13.51	99.2	0.64	3.93	246,817,976	236,917,289	0.96	28
LP97091	zoo	Loro Parque	16.55	99.0	0.66	2.67	270,656,048	258,046,972	0.95	31
LP97090	zoo	Loro Parque	16.96	96.6	0.63	5.81	231,761,348	221,943,054	0.96	26
LP97031	zoo	Loro Parque	24.38	99.3	0.66	6.70	268,129,884	256,737,602	0.96	31
LP97019	zoo	Loro Parque	24.39	99.1	0.64	7.24	309,771,318	296,302,089	0.96	36
LP97010	zoo	Loro Parque	24.41	99.3	0.64	3.65	231,412,614	223,412,812	0.97	27
LP97078	zoo	Loro Parque	28.61	99.5	0.64	4.48	246,714,716	236,658,624	0.96	29
LP97071	zoo	Loro Parque	35.20	99.6	0.63	5.51	258,475,514	248,671,374	0.96	30
Quintus	zoo	Zoo Zurich	4.83	99.8	0.64	9.34	228,787,248	218,170,798	0.95	26
Nils	zoo	Zoo Zurich	7.48	99.9	0.63	5.90	271,819,610	255,555,678	0.94	31
Noah	zoo	Zoo Zurich	7.72	99.8	0.65	7.52	263,622,330	247,997,431	0.94	30
Jeremy	zoo	Zoo Zurich	11.77	99.8	0.62	5.57	286,840,628	270,161,303	0.94	32
Ferdinand	zoo	Zoo Zurich	12.85	99.8	0.62	4.72	331,813,222	308,403,938	0.93	37
Engu	zoo	Zoo Zurich	13.72	99.9	0.61	9.12	254,438,230	242,253,129	0.95	29
Emil	zoo	Zoo Zurich	16.76	99.8	0.62	6.13	191,547,554	182,378,525	0.95	21
Bruno	zoo	Zoo Zurich	16.83	99.8	0.64	2.39	271,274,620	257,582,919	0.95	31
Seppi	zoo	Zoo Zurich	25.85	99.8	0.62	4.23	286,687,066	268,465,595	0.94	32
Falk	zoo	Zoo Zurich	29.33	99.7	0.62	8.94	227,744,980	217,046,796	0.95	26

Supplementary Table S4

-log2(FDR p-value)	FDR p-value	Pathway	DMRs	Total	Genes associated with DMR
**12.95**	**0.00013**	**Signal transduction**	52	(2597)	ARFGAP2, ARHGAP25, ARHGEF7, ARHGEF9, ARL4C, ATP2A3, BUB1, CAVIN1, CCT2, COL27A1, DHRS3, DNAL4, DOCK11, EIF4EBP1, FAM83D, FARP2, FGD3, FGFR3, FRS2, GFRA2, GRM4, HRH3, KSR2, LAMB3, MAMLD1, MAPKAP1, MDM2, MFNG, MYO9A, NCOR2, NR3C1, NRG1, NRTN, PAG1, PARD6A, PDE7B, PDE8B, PDGFRA, PPP1R12B, SCFD1, SH3GL1, SH3KBP1, STRN, TBL1X, TLE4, TNS4, TRHR, USP7, VWF, WNT5B, WWOX, ZNF512B
**11.79**	**0.00028**	**MAPK family signaling cascades**	18	(588)	EML4, MDM2, STRN, NCOR2, FGFR3, TBL1X, FIP1L1, MAPKAP1, NRG1, GFRA2, NRTN, FRS2, PDGFRA, LMNA, MAMLD1, VWF, LMO7, KSR2
		Diseases of signal transduction by growth factor receptors and second messengers	*16*	*(458)*	*EML4, MDM2, STRN, NCOR2, FGFR3, TBL1X, FIP1L1, MAPKAP1, NRG1, FRS2, PDGFRA, LMNA, MAMLD1, VWF, LMO7, KSR2*
		MAPK1/MAPK3 signaling	*8*	*(286)*	*FGFR3, NRG1, GFRA2, NRTN, FRS2, PDGFRA, VWF, KSR2*
		RAF/MAP kinase cascade	*8*	*(280)*	*FGFR3, NRG1, GFRA2, NRTN, FRS2, PDGFRA, VWF, KSR2*
		MAPK family signaling cascades	*8*	*(325)*	*FGFR3, NRG1, GFRA2, NRTN, FRS2, PDGFRA, VWF, KSR2*
**10.35**	**0.00077**	**Signaling by EGFR**	5	(59)	SH3GL1, SH3KBP1, FAM83D, PAG1, ARHGEF7
		Signaling by EGFR	*5*	*(53)*	*SH3GL1, SH3KBP1, FAM83D, PAG1, ARHGEF7*
		EGFR downregulation	*3*	*(31)*	*SH3GL1, SH3KBP1, ARHGEF7*
		GAB1 signalosome	*1*	*(17)*	*PAG1*
		Negative regulation of MET activity	*2*	*(21)*	*SH3GL1, SH3KBP1*
**9.72**	**0.00119**	**Notch signaling**	4	(38)	NCOR2, TBL1X, MAMLD1, MFNG
**9.60**	**0.00129**	**PI5P, PP2A and IER3 regulate PI3K/AKT signaling**	7	(135)	MDM2, STRN, FGFR3, MAPKAP1, NRG1, FRS2, PDGFRA
		Negative regulation of the PI3K/AKT network	*5*	*(113)*	*STRN, FGFR3, NRG1, FRS2, PDGFRA*
		PI5P, PP2A and IER3 regulate PI3K/AKT signaling	*5*	*(106)*	*STRN, FGFR3, NRG1, FRS2, PDGFRA*
		PI3K/AKT signaling in cancer	*7*	*(104)*	*MDM2, STRN, FGFR3, MAPKAP1, NRG1, FRS2, PDGFRA*
		Constitutive signaling by aberrant PI3K in cancer	*5*	*(78)*	*STRN, FGFR3, NRG1, FRS2, PDGFRA*
**9.08**	**0.00185**	**Signaling by PDGFR in disease**	3	(20)	STRN, FIP1L1, PDGFRA
		Signaling by PDGFRA extracellular domain mutants	*1*	*(12)*	*PDGFRA*
		Signaling by PDGFRA transmembrane, juxtamembrane and kinase domain mutants	*1*	*(12)*	*PDGFRA*
		Signaling by cytosolic PDGFRA and PDGFRB fusion proteins	*2*	*(3)*	*STRN, FIP1L1*
		Signaling by PDGFR in disease	*3*	*(20)*	*STRN, FIP1L1, PDGFRA*
**8.98**	**0.00198**	**Signaling by receptor tyrosine kinases**	15	(535)	COL27A1, FGFR3, MAPKAP1, WWOX, NRG1, SH3GL1, SH3KBP1, DNAL4, TNS4, FAM83D, FRS2, PDGFRA, PAG1, ARHGEF7, LAMB3
**8.00**	**0.00391**	**Circadian clock**	5	(86)	NR3C1, TBL1X, HMOX1, CRTC1, CRY1
		BMAL1:CLOCK, NPAS2 activates circadian gene expression	*1*	*(27)*	*TBL1X*
		Circadian clock	*4*	*(70)*	*NR3C1, TBL1X, CRTC1, CRY1*
		RORA activates gene expression	*1*	*(1)*	*TBL1X*
		Heme signaling	*3*	*(48)*	*TBL1X, HMOX1, CRTC1*
**7.94**	**0.00407**	**Transmission across chemical synapses**	12	(410)	SLC6A13, MDM2, GLUL, NRG1, NRXN1, KCNA3, KCNH5, GRIK3, GRIK4, CAMK1, ARHGEF7, ARHGEF9
		Neurotransmitter receptors and postsynaptic signal transmission	*7*	*(205)*	*MDM2, NRG1, GRIK3, GRIK4, CAMK1, ARHGEF7, ARHGEF9*
		Transmission across chemical synapses	*9*	*(270)*	*SLC6A13, MDM2, GLUL, NRG1, GRIK3, GRIK4, CAMK1, ARHGEF7, ARHGEF9*
		Neuronal system	*12*	*(410)*	*SLC6A13, MDM2, GLUL, NRG1, NRXN1, KCNA3, KCNH5, GRIK3, GRIK4, CAMK1, ARHGEF7, ARHGEF9*
**7.91**	**0.00416**	**RHOU GTPase cycle**	4	(54)	MYO9A, ZNF512B, PARD6A, ARHGEF7
		RHOV GTPase cycle	*4*	*(38)*	*MYO9A, ZNF512B, PARD6A, ARHGEF7*
		RHOU GTPase cycle	*2*	*(40)*	*PARD6A, ARHGEF7*
**7.67**	**0.00491**	**Signaling by ALK in cancer**	5	(91)	EML4, MDM2, STRN, FRS2, LMO7
		ALK mumants bind TKIs	*2*	*(12)*	*EML4, STRN*
		Signaling by ALK fusions and activated protein mutatns	*5*	*(91)*	*EML4, MDM2, STRN, FRS2, LMO7*
		Signaling by ALK in cancer	*5*	*(91)*	*EML4, MDM2, STRN, FRS2, LMO7*
